# Hydromorphone Prescription for Pain in Children—What Place in Clinical Practice?

**DOI:** 10.3389/fped.2022.842454

**Published:** 2022-04-25

**Authors:** Frédérique Rodieux, Anton Ivanyuk, Marie Besson, Jules Desmeules, Caroline F. Samer

**Affiliations:** ^1^Division of Clinical Pharmacology and Toxicology, Department of Anesthesiology, Pharmacology and Intensive Care, Geneva University Hospitals, Geneva, Switzerland; ^2^Faculty of Medicine, University of Geneva, Geneva, Switzerland; ^3^Institute of Pharmaceutical Sciences of Western Switzerland (ISPSO), School of Pharmaceutical Sciences, University of Geneva, Geneva, Switzerland

**Keywords:** hydromorphone, opioids, children, safety, pain

## Abstract

While morphine is the gold standard treatment for severe nociceptive pain in children, hydromorphone is increasingly prescribed in this population. This review aims to assess available knowledge about hydromorphone and explore the evidence for its safe and effective prescription in children. Hydromorphone is an opioid analgesic similar to morphine structurally and in its pharmacokinetic and pharmacodynamic properties but 5–7 times more potent. Pediatric pharmacokinetic and pharmacodynamic data on hydromorphone are sorely lacking; they are non-existent in children younger than 6 months of age and for oral administration. The current data do not support any advantage of hydromorphone over morphine, both in terms of efficacy and safety in children. Morphine should remain the treatment of choice for moderate and severe nociceptive pain in children and hydromorphone should be reserved as alternative treatment. Because of the important difference in potency, all strategies should be taken to avoid inadvertent administration of hydromorphone when morphine is intended.

## Introduction

Pain is an important public health problem. In pediatrics, it is the most common symptom in the emergency setting (1) and can affect up to 50–75% of children during their hospitalization (2).

Although pain management in children has improved dramatically, many challenges remain and prescribing analgesics in this population can be complex for several reasons. First, due to ontogeny, the response to most medications when used in children, especially neonates, differs from that of adults. Due to the physiological maturation and development of their different organs, transporter and enzyme systems, the pharmacokinetics (PK) and pharmacodynamics (PD) of drugs are different in children compared to adults. All stages of PK are affected: the degree of protein binding is usually decreased, the volume of distribution (Vd) of many drugs is modified according to changes in body composition, and the activities of many enzymes and drug transporters involved in drug metabolism and disposition are significantly decreased during the first years of life which impacts not only hepatic and renal clearance, but also their passage through biological barriers such as the blood-brain barrier (BBB). Besides this, the capacity of the target organ to respond to medications may also differ in children compared to adults. For analgesics in particular, assessment of their effect may be limited in young children with little or no verbal communication, leading to a risk of ineffectiveness or intoxication. Finally, the therapeutic choice is limited by the lack of efficacy and safety data and approved indications for many analgesic drugs.

Despite these obstacles, effective pain management in children is essential, not only for the child's comfort, daily life and activities but also to avoid development of a chronic pain syndrome related to central sensitization and altered quality of life in the medium and long term (3–7). Pain management should be a multimodal approach, including medications from different analgesic classes, procedural interventions and rehabilitation. Pharmacological treatment in children still follows the World Health Organization's three-step approach, i.e., non-opioids, non-steroidal anti-inflammatory drugs (NSAIDs) and paracetamol, for mild nociceptive pain; non-opioids and weak opioids, such as tramadol and codeine, for moderate nociceptive pain; and non-opioids and strong opioids for severe nociceptive pain. A two-step approach is increasingly advocated today: NSAIDs and paracetamol for mild pain, and non-opioids and strong opioids for severe pain, omitting, weak opioids (8). Despite the lack of formal comparisons between the two-step and three-step treatment in children, the risks associated with strong opioids appears to be more acceptable than the uncertainty associated with the variability in drug response observed with codeine and tramadol (9, 10).

Among strong opioids, morphine is the one for which most data are available in children. Morphine has been shown to be effective and safe when used appropriately in children (11, 12). It can be used in children of all ages and is available in a variety of dosage forms (13). Morphine is thus the gold standard for treating severe pain in children.

Hydromorphone is another strong opioid which can be administered both intravenously (IV) and orally, and whose administration appears to be increasing in children of all ages, including infants (14). We are also seeing this increase in our practice, and although some prescribers claim that nausea-vomiting and pruritus are less common with hydromorphone, the rational for prescribing hydromorphone in children instead of morphine is not always known.

In order to better understand whether hydromorphone is a safe option and an alternative to morphine for severe pain treatment in children, this article aims to review the available literature on hydromorphone in children, particularly on its PK and safety.

Relevant articles in the PubMed and EMBASE databases, published until September 2021, were identified using the following keywords: “neonates”, “infant”, “children”, “pediatric”, “hydromorphone”, “pharmacokinetics”. The following article types were eligible: original articles, PK/PD reviews, epidemiologic studies and case reports. Our search was limited to English-language studies published in peer-reviewed journals. Additional publications were identified by reviewing references of these original. The Swiss (SwissmedicInfo), American (Food and Drug Administration, FDA), English (British National Formulary for children, BNFc) and French (Vidal) summary of product characteristics were consulted.

## Discussion

### Hydromorphone

Hydromorphone is a semi-synthetic opioid analgesic with potent mu-agonist activity. It was first marketed in the U.S. in the 1920s.

**Figure 1:**
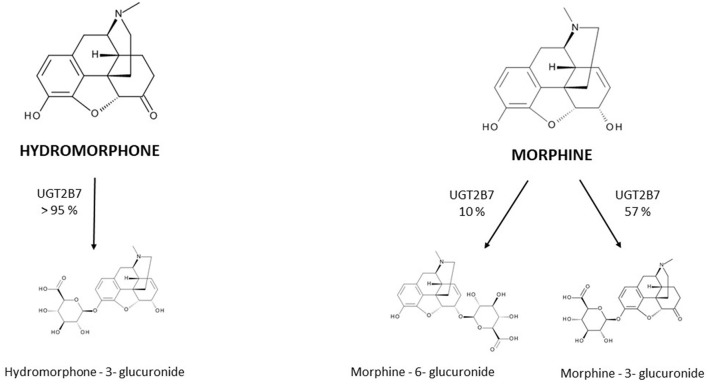
Metabolism of hydromorphone and morphine into their main metabolites. UGT = uridine 5′-diphospho-glucuronosyltransferase.

Hydromorphone is structurally very similar to morphine (Figure 1); differing by the presence of a 6-keto group and the hydrogenation of the double bond at the 7–8 position of the molecule (15).

It is marketed in various formulations, including injection solution, (extended-) tablet, oral solution and suppository.

### Pharmacokinetics

The PK of hydromorphone is well described in adults. In this population, after oral administration hydromorphone is rapidly absorbed and is subject to a significant first-pass effect, leading to a mean systemic oral bioavailability of 32% with wide interindividual variation (17–62%) (16–19); the maximum serum concentration (Cmax) is reached in less than an hour for immediate-release forms. After intranasal administration of the injection solution, a bioavailability of 50–60% is described (20, 21). Rectal administration has also been evaluated in small studies (*n* < 10) and has been found to have a bioavailability of around 30% (10–65%) (17, 18). Hydromorphone is a lipophilic molecule with a limited protein binding capacity of 7–19%, and its apparent Vd is relatively small, estimated to be approximately 1.22-4 L/kg. It is extensively metabolized (>95%) in the liver by uridine 5'-diphospho-glucuronosyltransferase 2B7 (UGT2B7) to hydromorphone-3-glucuronide (H3G), which has no intrinsic pain-relieving effects but is thought to have neuroexcitatory adverse effects (22–25). Other metabolites are dihydromorphine (<1%), dihydroisomorphine (1%) and their glucuronides. The involvement of P-glycoprotein (P-gp) in hydromorphone transport is not clearly established to date and rapid membrane crossing, including the BBB, is observed due to the liposolubility of hydromorphone (26).

Hydromorphone is therefore not only structurally but also pharmacokinetically very similar to morphine. Both molecules have interindividual variation in oral bioavailability, undergo glucuronidation primarily by UGT2B7, are metabolized to a 3-glucuronide metabolite and are eliminated by renal route. Response to both hydromorphone and morphine treatments may be influenced by polymorphisms in the μ-receptor gene (*OPRM1*), as well as drug interactions involving the UGT2B7 (27, 28). In adults, their key PK parameters such as bioavailability, Vd and half-life, are comparable (Table 1). Their differences are mainly for hydromorphone (i) a less well-defined role of the P-gp efflux transporter in the BBB penetration and brain disposition (26, 31, 32) and (ii) the lack of active 6-glucuronide metabolite formation. For this last reason, hydromorphone use in patients with severe renal impairment is often viewed as a safer alternative to morphine. However, evidence of a larger safety margin in renal failure is limited and both molecules should be used with caution due to the accumulation of their 3-glucunonide metabolites.

**Table 1:** Main PK parameters of hydromorphone and morphine.

	**Bioavailability** **(%)**	**Tmax** **(h)**	**Protein binding** **(%)**	**Volume of** **distribution** **(L/kg)**	**Metabolism** **(Main pathway)**	**Main metabolites**	**Excretion** **(Main pathway)**	**Half-life** **(h)**
Hydromorphone	17–62	1	7–19	1.2–4.0	UGT2B7	H3G	Kidney	1.5–4.0
Morphine	20–40	1	20–35	3–4	UGT2B7	M6G (10–15 %) + M3G (50–57 %)	Kidney	2.0–4.0

*PK, Pharmacokinetics; Tmax, Time to reach maximum concentration; h, Hour; L, Liter; kg, Kilogram; UGT, Uridine 5'-diphospho-glucuronosyltransferase; M6G, Morphine-6-glucuronide; M3G, Morphine-3-glucuronide; H3G, Hydromorphone-3-glucuronide; T1/2, Plasma half-life time (16, 17, 19, 29, 30)*.

All mentioned data and observations are from studies in adults. In young children, ontogenic changes and other age-related differences can significantly alter the PK of drugs, for both morphine and hydromorphone, making simple extrapolation of adult data inappropriate.

Regarding morphine, the effect of ontogeny is well described. It is thus known that the estimated oral bioavailability is higher in very young infants than in healthy adults (33). Data on the ontogeny of UGTs are scarce, but UGT2B7 isoenzyme activity is reduced at birth and seems to reach adult activity levels between 1 and 12 months of age (34). Consistent with the immaturity of hepatic glucuronidation by UGT2B7, the limited ability of neonates to glucuroconjugate morphine is well documented (35, 36). Renal function which is represented by glomerular filtration rate (GFR) changes quickly with the maturation of young children, reaching adults' capacity between 6–12 months (37). Morphine clearance is typically slower in infants and approaches adult values by 6 months of age (38); therefore, the half-life is longer in the earliest stages of life and decreases as metabolic pathways develop (39). The neonatal BBB shows a lower barrier capacity than in adults, due to lower expression of barrier-related proteins and lower function of the P-gp, which reaches adult activity between 3 and 6 months of age (32, 40). This increase in permeability contributes, amongst others factors, to the increased sensitivity of neonates and young infants to the central depressant effects of morphine (32, 41).

As hydromorphone is pharmacokinetically very similar to morphine, the same changes, as described above, could be expected. However, data on the PK of hydromorphone in children are much sparser. We found only two studies that evaluated hydromorphone PK in the pediatric population (42, 43). The first study by Collins et al. included 10 children randomly assigned to receive either morphine or hydromorphone by patient-controlled analgesia (PCA) (mean ages 13.7 and 15.3 years respectively) to manage mucositis pain. Blood samples were drawn 2, 4, and 6 h after the start of a continuous infusion and only clearance was determined (51.7 mL/min/kg; range, 28.6–98.2). In the second, more recent prospective study by Balyan, 34 children [mean age 13.5 (4–18 years), bodyweight 56.7 (23–89.6 kg)] undergoing elective surgery (spine, neurological, or abdominal surgery) were treated with IV hydromorphone boluses followed by PCA. The PK profile was determined by measuring hydromorphone concentrations before and 3, 10, 30, and 90 min after the first dose and by using nonlinear mixed-effects modeling. The study demonstrated that body weight was a significant covariate for clearance while gender, race and type of surgery were not. Vd was comparable to the one described in prior adult studies (33 L/70kg vs. 3.35–42.7 L/70kg) and clearance value was smaller (0.738 L/min/70kg vs. 1.02-1.81 L/min/70kg) (17, 44, 45). Therefore, these two studies give us no information regarding other relevant PK properties, such as bioavailability or time to reach maximum concentration (Tmax), and above all, they provide no PK data for young children, particularly for infants younger than 6–12 months in whom the effect of ontogeny is the most expected.

### Pharmacodynamics

Hydromorphone is a non-selective opioid receptor agonist with predominant affinity for μ-receptors and lower affinity for k- and d-receptors.

The efficacy and safety of hydromorphone are documented in adults, regardless of route of administration (46–48). As with all opioids, there is a large interindividual variability in the dose-efficacy-toxicity relationship. The “appropriate” dose for a given patient varies depending on many factors, including individual factors (gender, weight, comorbidities, organ function, previous exposure to opioids, ontogeny...) as well as genetic and environmental factors (comedications, diet...). The recommended initial dose often needs to be adjusted according to individual pain intensity, efficacy and occurrence of adverse drug reactions (ADRs). The most commonly described ADRs of hydromorphone are related to its binding to the μ-opioid receptors and are therefore, at equianalgesic doses, similar to the ADRs of other opioids. They consist mainly of dizziness, nausea, confusion, drowsiness, vomiting, constipation, pruritus and dry mouth; more rarely, respiratory depression and impaired consciousness. In adults, no study has demonstrated a different ADR profile, including nausea and pruritus, between hydromorphone and morphine at equianalgesic doses (48–50). The higher affinity for μ-receptors makes hydromorphone a more potent analgesic than morphine. The equianalgesic dose ratio between parenteral hydromorphone and morphine, calculated from adult studies, is approximately 1:5–7 (48, 51, 52). The same is true for the oral equianalgesic dose (52, 53).

In children, the efficacy of hydromorphone to treat perioperative pain has been demonstrated in a small number of studies when administered IV, either in bolus, continuous or PCA (14, 43, 54–60). The efficacy of epidural administration has also been established (61–67) and a recent study showed the efficacy of intranasal administration (68). Hydromorphone appears to be as effective as morphine, fentanyl and sufentanyl. These studies, whatever the route of administration, primarily included children and adolescents. Only two of them included infants (54, 59). These studies showed good tolerance of hydromorphone in infants, children and adolescents. Adverse effects were comparable to those described in adults, mainly nausea, vomiting and pruritus (14, 43, 57, 58, 60–68).

Spénard et al. recently published an excellent systematical review that sought to compare the efficacy and safety of hydromorphone and morphine in children (69). Among 754 abstracts reviewed, they found only four randomized controlled trials that compared the PD of hydromorphone and morphine in children (43, 56, 57, 61). In three of them, treatment was administered IV (43, 56, 57), in bolus or PCA doses, with equianalgesic dose ratio ranging from 5:1–7:1. The last of the four studies involved epidural administration and none involved oral administration. More than 150 children and teenagers were included, but none were younger than 3 years of age. Two of the studies involving IV administration showed no statistically significant difference in pain scores with morphine compared with hydromorphone. Only the study by Chen et al. showed that significantly more patients in the morphine group required extra fentanyl for pain relief, however with no significant difference in analgesia satisfaction score between the two groups (56). The three studies reporting the use of the IV route showed no significant difference in adverse effects, including nausea, sedation and pruritus (43, 56, 57). Only the study in which hydromorphone and morphine were administered epidurally found a higher incidence of pruritus related to the use of morphine (8% for hydromorphone vs 35% for morphine) (61). These findings should be taken with caution, as the relatively low (8%) incidence of pruritus on hydromorphone described in this study does not corroborate with the 30% to almost 70% incidence of pruritus reported in other studies (64, 65, 67).

Regarding the hydromorphone to morphine equianalgesic dose ratio, only one pediatric study has assessed the equipotence of hydromorphone vs. morphine (43). In this double-blind three-period crossover study, 10 children (mean ages 13.7 and 15.3 years for group 1 and 2, respectively) with mucositis pain received morphine or hydromorphone by PCA in a 7:1 ratio. Analysis of variance of total opioid doses indicated that patients used 27% more hydromorphone than expected, suggesting a mean equipotence of 5:1, comparable to that derived from adult's studies. No study has determined the equianalgesic dose ratio between oral hydromorphone and morphine in children and the same ratio is used in children of all ages, including infants, without taking into account the ontogenic considerations described above.

### Dosing Recommendations

Marketing authorization for hydromorphone administration in children is restricted and varies from country to country (Table 2). Due to the few studies available on its epidural or intranasal administration, the only routes of administration approved by the majority of national regulatory authorities are oral, SC and IV injection (bolus, continuous or via PCA). In the United States (US), there is no labeled indication in children, regardless of the route of administration.

**Table 2:** Labeled authorization (non-exhaustive list).

**Country**	**Authorized routes of administration in adults**	**Authorized routes of administration in children**	**Therapeutic indications**
US labeled authorization	IV, SC and IM (bolus injection)	**No authorization**	moderate to severe pain
	Rectal	**No authorization**	moderate to severe pain
	Oral	**No authorization**	moderate to severe pain
Swiss labeled authorization	IV and SC (bolus injection, infusion)	from **1** year of age	moderate to severe pain
	PCA (IV and SC)	from **12** years of age	moderate to severe pain
	Oral	from **12** years of age	moderate to severe pain
UK labeled authorization	IV and SC (bolus injection, infusion)	from **12** years of age	severe pain in cancer
	PCA (IV and SC)	from **12** years of age	severe pain in cancer
	Oral	from **12** years of age	severe pain in cancer
French labeled authorization	Oral	from **7** years of age	severe pain in cancer

*IV, intravenous; SC, subcutaneous; IM, intramuscular; PCA, patient-controlled analgesia*.

Various international expert opinions and formularies (70–79) have issued dosing recommendations for IV and oral hydromorphone in children. These recommendations vary widely and their scientific evidence is not described (Table 3).

**Table 3:** Examples of pediatric dosing recommendations.

**(A) IV bolus**				
**Source**	**“Age category” as mentioned in the referenced source**	**Recommended starting dose**		
		**Dose** **(mg/kg/dose)**	**Dose** **(mg/dose)**	**Interval** **(h)**
FDA	-	-	-	-
Swissmedicinfo (70)	≥12 months and <12 years	0.015	-	3–4
	>12 years and <50 kg	0.015	-	3–4
	>12 years and >50 kg	-	1–1.5	3–4
BNFc (72)	-	-	-	-
Kraemer and Rose (73)	Infants and children	0.010–0.020	-	3–4
Zernikow et al. (74)	>6 months and >10 kg	0.010 (max 0.5 mg/dose)	-	3
Friedrichsdorf and Kang (75)	Children ≤ 50 kg	0.015	-	3–4
	Children >50 kg	-	1–1.5	3–4
Berde and Sethna (76)	<6 months	^*^	^*^	^*^
	>6 months and <50 kg	0.020	-	2–4
	>6 months and ≥50 kg	-	1	2–4
Lexicomp (77)	Infants >6 months and >10 kg Children <50 kg Children ≥50 kg	0.010–0.015 0.015 -	- - 0.2–0.6	3–6 3–6 2–4
Pediatrics, in Micromedex (78)	≥6 months and <50 kg	0.010–0.020 (max 0.5 mg/dose)	-	3–4
	≥6 months and ≥50 kg	-	1–1.5	3–4
Kinderformularium (79)	≥1 month and <10 kg	0.003–0.005	-	3–4
	≥1 month and <50 kg	0.010–0.015	-	3–4
	≥1 month and ≥50 kg	-	1.0–1.5	3–4

*IV, intravenous; -, no data*.

* *The author recommends in a comment note “In infants under six months, initial per-kilogram doses should begin at roughly 25 percent of the per-kilogram doses recommended” in older infants (76)*.

**Table 3B:** IV, Continuous infusion.

**Source**	**“Age category” as mentioned in the referenced source**	**Recommended starting dose**	
		**Dose** **(mg/kg/h)**	**Dose** **(mg/h)**
FDA	-	-	-
SwissmedicInfo (70)	≥12 months and <12 years	0.005	-
	>12 years and <50 kg	0.005	-
	>12 years and >50 kg	0.004	0.15–0.45
BNFc (72)	-	-	-
Kraemer and Rose (73)	Infants and children	0.003–0.005	-
Zernikow et al. (74)	>6 months and >10 kg	0.005 (max. 0.2 mg/h)	-
Friedrichsdorf and Kang (75)	Children ≤ 50 kg	0.003−0.005	-
	Children >50 kg	-	-
Berde and Sethna (76)	<6 months	^*^	^*^
	>6 months and <50 kg	0.006	-
	>6 months and ≥50 kg	-	0.3
Lexicomp (77)	>6 months and >10 kg	0.003–0.005 (max 0.2 mg/h)	-
	Children <50 kg	0.003–0.005 (max 0.2 mg/h)	-
	Children ≥50 kg	-	0.3
Pediatrics, in Micromedex (78)	≥6 months and <50 kg	0.003–0.006 (max 0.2 mg/h)	-
	≥6 months and ≥50 kg	-	0.3
Kinderformularium (79)	≥1 month and <10 kg	0.001–0.002	-
	≥1 month and <50 kg	0.003–0.005	-
	≥1 month and ≥50 kg	0.003–0.005 (max 0.45 mg/h)	-

*IV, intravenous; -, no data*.

* *The author recommends in a comment note “In infants under six months, initial per-kilogram doses should begin at roughly 25 percent of the per-kilogram doses recommended” in older infants (76)*.

**Table 3C:** PCA.

**Source**	**“Age category” as mentioned in the referenced source**	**Recommended starting dose**				
		**Demand dose (mg/kg)**	**Demand dose (mg)**	**Lockout interval (min)**	**Basal infusion (mg/kg/h)**	**Rescue dose (mg/kg)**
FDA	-	-		-	-	-
SwissmedicInfo (70)	≥12 months and <12 years	-	-	-	-	-
	>12 years and <50 kg	-	-	-	-	-
	>12 years and >50 kg	-	0.2	5–10	-	-
BNFc (72)	-	-	-	-	-	-
Kraemer and Rose (73)	Infants and children	0.004		8–10	0–0.004	0.01
Zernikow et al. (74)	>6 months and >10 kg	0.004 (max. 0.2 mg)	-	-	-	-
Friedrichsdorf and Kang (75)	-	-	-	-	-	-
Berde and Sethna (76)	-	-	-	-	-	-
Lexicomp (77)	Children ≥5 years and <50 kg	0.003–0.004	-	6–10	0–0.004	–
	Children ≥50 kg	0.1–0.2		6		
Pediatrics, in Micromedex (78)	≥6 years	0.004 (max. 0.2 mg)	-	5–10	0.0014–0.004	0.01
Kinderformularium (79)	≥1 month and <10 kg	-	-	-	-	-
	≥1 month and <50 kg	0.003-0.004	-	5–10	0.003–0.005	-
	≥1 month and ≥50 kg	-	0.2	5–10	0.003–0.005	-

*PCA, Patient-controlled analgesia; -, no data*.

**Table 3D:** Oral, immediate release.

**Source**	**“Age category” as mentioned in the referenced source**	**Recommended starting dose**		
		**Dose (mg/kg)**	**Dose (mg)**	**Interval (h)**
FDA	-	-	-	-
SwissmedicInfo (71)	≥12 years	-	1.3–2.6	4
BNFc (72)	≥12 years	-	1.3	4
Kraemer and Rose (73)	Infants and children	0.04–0.08	-	4
Zernikow et al. (74)	>6 months and >10 kg	0.03 (max 1.3 mg)	-	4
Friedrichsdorf and Kang (75)	Children ≤ 50 kg	0.03–0.06	-	3–4
	Children >50 kg	-	1- 2	3–4
Berde and Sethna (76)	<6 months	^*^	^*^	^*^
	>6 months and <50 kg	0.04–0.08	-	3–4
	>6 months and ≥50 kg	-	2–4	3–4
Lexicomp (77)	Infants >6 months and >10 kg	0.03	-	4
	Children and adolescents <50 kg	0.03–0.08	-	3–4
	Children and adolescents ≥50 kg	-	1–2	3–4
Pediatrics, in Micromedex (78)	≥6 months and 10-50 kg	0.03–0.08 (max 1.3 mg)	-	3–4
	≥6 months and ≥50 kg	-	1–4	3–4
Kinderformularium (79)	≥1 month and <10 kg	0.01–0.02	-	3–4
	≥1 month and ≥10 kg	0.03–0.08 (max 2.6 mg)	-	3–4

*-, no data*.

* *The author recommends in a comment note “In infants under six months, initial per-kilogram doses should begin at roughly 25 percent of the per-kilogram doses recommended” in older infants (76)*.

As summarized in Table 3, the majority of IV recommendations tend to agree on a dosage of 0.01–0.02 mg/kg/dose every 3–4 h or 0.003–0.006 mg/kg/h for continuous infusion regardless of patient's age, but most often specifying an age older than 6 months or a weight higher than 10 kg. The Dutch Kinderformularium, a database developed by the Dutch Knowledge Centre for Pediatric Pharmacotherapy (Nederlands Kenniscentrum Farmacotherapie bij Kinderen: NKFK), available online at www.kinderformularium.nl, provides specific dosing for young infants, distinguishing between infants under or over 10 kg. They recommend a much lower dosage in infants under 10 kg: 0.003–0.005 mg/kg/dose every 3–4 hours (Kinderformularium.nl). Berde et al., in a small comment note under their guidelines table, specify that “in infants under 6 months, initial per-kilogram doses should begin at roughly 25 percent of the per-kilogram doses recommended” in older children (76).

In line with the oral bioavailability of hydromorphone described in adults, the most commonly recommended oral pediatric dose is 0.03–0.06 mg/kg/dose every 3–4 h. For young infants under 10 kg, the Dutch Kinderformularium recommends a dosage of 0.01–0.02 mg/kg/dose every 3–4 h. As with IV administration, Berde et al. recommend that “in infants under 6 months, initial per-kilogram doses should begin at roughly 25 percent of the per-kilogram doses recommended” in older children (76).

## Conclusion/Recommandation

Hydromorphone is a morphine derivative with significantly greater analgesic potency than morphine. Except for its higher potency, hydromorphone does not differ substantially from morphine in PK, analgesic efficacy and ADRs.

Available data on the use of hydromorphone in children is very limited and non-existent for oral administration and for children under 6 months of age. Current data do not support an advantage of hydromorphone over other opioids, particularly over morphine, in terms of both efficacy and safety. Despite its increasing use, until more studies examining the use of hydromorphone are available in children, morphine remains the drug with the strongest evidence of efficacy and safety and should remain the opioid of first choice in the pediatric population for the management of severe nociceptive pain. IV hydromorphone is a valuable alternative when morphine is poorly tolerated.

The prescriber should be aware that the use of hydromorphone in children is an off-label prescribing in most situations. The prescriber should have specific knowledge and experience with this drug in children and should also take into account the conditions that the European Academy of Paediatrics and the European Society for Perinatal and Developmental Paediatrics Pharmacology (ESDPPP) have recently defined to facilitate rational and safe prescribing of off-label drugs (80). When prescribing hydromorphone, whatever the route of administration, in young infants under 6 months or 10 kg, dosing should consider the possible impact of ontogeny, such as decreased clearance and increased permeability of the BBB. The simple weight-adjusted dosing recommendation used in older children is probably not safe enough, and to minimize the risk of ADR, a lower starting dose, as proposed by the Dutch Kinderformularium and Berde et al., seems warranted. Great caution is required when administering an oral form to infants and young children due to the lack of data. Attention should be paid to the choice of age-adapted dose formulation. As with other opioids, regular and close assessments of efficacy and ADRs are essential and should allow prompt dosage adjustments in children of all ages. Adverse events should be reported to the national pharmacovigilance agencies.

Because of its higher potency, inadvertent prescription and administration of hydromorphone when morphine is intended can have severe, potentially fatal, consequences, in particular in children. Caregivers prescribing or administering hydromorphone should be aware of this difference in potency, and standard strategies such as Tall Man lettering (which uses capital letters to help differentiate between look-alike drug names) and color coding should be implemented.

Further clinical studies describing the PK and PD of hydromorphone in children are needed. Given the real-world difficulty of including children in PK studies, physiologically-based pharmacokinetic (PBPK) modeling may help acquire data on the influence of age-dependent physiological differences on hydromorphone PK.

## Author Contributions

FR wrote the manuscript. CS, AI, MB, and JD revised the manuscript and approved the final version. All authors contributed to the article and approved the submitted version.

## Funding

Open access funding was provided by the University of Geneva.

## Conflict of Interest

The authors declare that the research was conducted in the absence of any commercial or financial relationships that could be construed as a potential conflict of interest.

## Publisher's Note

All claims expressed in this article are solely those of the authors and do not necessarily represent those of their affiliated organizations, or those of the publisher, the editors and the reviewers. Any product that may be evaluated in this article, or claim that may be made by its manufacturer, is not guaranteed or endorsed by the publisher.
