# Erratum: Hydromorphone prescription for pain in children—What place in clinical practice?

**DOI:** 10.3389/fped.2023.1186148

**Published:** 2023-03-28

**Authors:** Frontiers Production Office

**Affiliations:** Frontiers Media SA, Lausanne, Switzerland

**Keywords:** hydromorphone, opioids, children, safety, pain

An Erratum on Hydromorphone prescription for pain in children—What place in clinical practice? By Rodieux F, Ivanyuk A, Besson M, Desmeules J and Samer CF. (2022) Front. Pediatr. 10:842454. doi: 10.3389/fped.2022.842454

An omission to the funding section of the original article was made in error. The following sentence has been added: “Open access funding was provided by the University of Geneva”.

The original version of this article has been updated.

